# Family Function and Self-esteem among Chinese University Students with and without Grandparenting Experience: Moderating Effect of Social Support

**DOI:** 10.3389/fpsyg.2017.00886

**Published:** 2017-05-30

**Authors:** Jingyu Shi, Lu Wang, Yuhong Yao, Na Su, Xudong Zhao, Chenyu Zhan

**Affiliations:** ^1^East Hospital, Tongji University School of MedicineShanghai, China; ^2^Student Counselling Center, Tongji UniversityShanghai, China; ^3^Medical School of Tongji UniversityShanghai, China; ^4^Pudong Mental Health CenterShanghai, China

**Keywords:** social support, self-esteem, family function, Chinese university students, grandparenting

## Abstract

This study examines the association between family function and self-esteem of Chinese university students with grandparenting experience, and explores the moderating effects of social support in this link. Two thousand five hundred thirty university students (1372 males and 1158 females) from a Chinese university completed the Perceived Social Support Scale, the Rosenberg’s Self-esteem Scale, and the Family Assessment Device (FAD). Six hundred and forty-five (25.69%) students reported grandparenting experience and they reported lower scores on self-esteem and social support than the students raised only by their parents. The grandparenting group scored higher on such dimensions of family functioning as Communication, Role, Affective Involvement, Affective Responsiveness, and General Family Function (GF) than their counterpart group. For both groups, self-esteem scores were positively correlated with social support scores, while negatively correlated with FAD all sub-scale scores. Hierarchical regression analysis showed that for the students with grandparenting experience the social support moderated the relationship between GF and self-esteem. When students reported a high level of social support, those with low GF score reported higher scores in self-esteem than those with low self-esteem. However, in case of low social support, there were no differences in self-esteem between groups with high and low GF scores. These findings suggest that social support plays a positive role to relieve the adverse impact of poor family function on self-esteem of the adolescents with grandparenting experience. In addition, the significance and limitations of the results will be discussed.

## Introduction

Since the 1980s, due to the radical development of the society resulting from China’s “reform and opening,” there were many significant changes within the Chinese family. One of the major changes was the introduction of grandparenting which became an important family rearing pattern. Grandparenting refers to the situation of grandparents rearing and educating their grandchildren. A nationwide survey in China has shown that in the largest cities, such as Beijing, Shanghai, and Guangzhou, among children under the age of six those accepting grandparenting accounted for more than 50% (Wu, 2010). Therefore, grandparenting has become an important regular condition of children’s growth in China. In the European countries and United States of America, the reasons for grandparenting are mainly based on the parents’ marital status (divorce, separation, premarital pregnancy, etc.) and economic reasons, while in China there are unique reasons for this phenomenon: first of all, Chinese traditional culture has formed strong and unique family ethics, as part of this moral code of conduct, grandparents view raising grandchildren as their responsibilities, and through this it is considered to obtain happiness and satisfaction. In addition, the young parents spend most of the time and energy on working or further studying, which means that they cannot fully take care of their children, therefore the children are handed over to the grandparents to be raised. Prevailing studies on grandparenting are mainly concerned with the impact on the physical and mental health of grandparents (Muller and Litwin, 2011; Grundy et al., 2012; Arpino and Bordone, 2014; Burn et al., 2014; Zhang and Han, 2016). Other research is focusing on the influence of grandparenting on the psychological and social development of the children, reporting a negative effect on their self-esteem. The children being raised by their grandparents show more psychological problems and difficulties with social adaptation in family and school life (Qi, 2011; Edwards and Daire, 2006; Li and Li, 2006; Zhang and Lu, 2017). Compared with children who were raised by their parents, they showed more emotional and behavior problems (Edwards, 2006; Edwards and Daire, 2006; Wang, 2007; Qi, 2011; Zhang and Lu, 2017), they were shy and had little skills in communicating with others (Fuller-Thomson and Minkler, 2005; Xin, 2010; Han and Guo, 2016). However, the main research objects in this study were pre-school or primary school children who currently accept grandparenting. Due to a lack of retrospective studies on the influence of grandparenting on adolescents and young adults, we raised the following question: Do children with a background of being brought up by their grandparents up to adolescence or adulthood show different levels of self-esteem compared to their peers without the experience of grandparenting?

Self-esteem refers to the evaluation and experience related to self-value, the perception of self ability as well as the acceptance of the whole self, which an individual obtains during the process of socialization (Rosenberg, 1965; Leary and MacDonald, 2005). According to Maslow (1987) a person with high self-esteem feels more confident, is more competent, and hence more productive; while an individual with low self-esteem often feels inferior, hopeless, even desperate and tends to get neurosis. An individual with high self-esteem has a high level of mental health status and self-harmony (Peng et al., 2013). Moreover, self-esteem could positively affect the general well-being (Shim et al., 2013; Tian et al., 2013). Self-esteem might also contribute to a higher success motivation (Pang et al., 2011). In addition, it might affect the ability to achieve goals or increase the ability to cope with problems, while low self-esteem may lead to avoidance (Hendy et al., 2003). Self-esteem can also affect decision-making, which may have an impact on the individual’s entire life (D’Amico and Cardaci, 2003). Self-esteem is one of the basic needs for all individuals especially in adolescence. College Students are in the transitional period from adolescents to adults, they inevitably experience great changes during the shift from middle school students to college freshmen. They should independently manage their college life and cope with challenges. Both family and society put high expectations on them, and at the same time they must adjust to the new environment. Although their intellectual and physical development has already been completed, their psychological development is not yet fully mature, and in addition their self-consciousness is still developing. Therefore the college students are easily to experience inner conflicts and ambivalence. Self-esteem as an important trait of the self-system has a positive impact on the mental health, personality development and social adaptation of the individual (Leary and MacDonald, 2005).

The formation and development of one’s self-esteem is not only the result of the socialization process, but also the product of the interaction with one’s sociocultural and familial environment as well as school education. Most of the research findings demonstrated that children’s self-concepts are related to parent-child relationships, and family characteristics (Brown et al., 2009). Brown et al. (2009) found that the interplay of familial relationships has an impact on a child’s developing sense of self: In the families that show more harmonious interactions, the children describe themselves as being more adventurous. However, it is more likely that in the families which experience high levels of discord the children view themselves as more fearful and less agreeable. The results of another study showed that the parents of the children with low self-esteem tend to punish the children severely, whereas in the group of the children with high self-esteem the family exhibited a more democratic way of interaction (Peng and Fan, 2007; Heaven and Ciarrochi, 2008). Various researchers examined the relationship between family function and self-esteem, they consistently found that there is a close relationship between self-esteem and family function, and the results have cross-cultural consistency: Wang et al. (2010) for example found that there is a significant correlation between the development of adolescent self-esteem and parental rearing styles. Other data showed that there is a significant positive correlation between a high level of family intimacy as well as emotional expression and a high level of self-esteem and self achievement of adolescents (Li et al., 2006); the extent of closeness of family members and the parental expectations might affect the self-esteem and self-concept of the college freshmen (Wu and Ye, 2009). However, grandparenting experience might affect the parent-child relationship. Li (2010) examined the parent-child relationship of college students with grandparenting experience during childhood and found that intimacy between parents and children was lower compared to those with non-grandparenting experience; the students felt distant from their parents and the parents did not trust in their children’s capability due to a lack of initiative communication between parents and children. Hence, it is necessary to explore whether and how the family function of the students with grandparenting experience influences their self-esteem? Compared with students solely raised by their parents, in case of existing factors, which ones do have a moderating effect on the relationship between family function and self-esteem of the students with grandparenting experience?

Social support can be defined as an individual’s perception or experience that their social network will provide effective emotional and substantial support during the times of need (Wills, 1991). The consistent finding in many researches show that social support is positively related to self-esteem (e.g., Barrera and Garrison-Jones, 1992; Wentzel, 1994; Sweeting and West, 1995; Goodwin et al., 2004; Birndorf et al., 2005; Zhang, 2007; Denissen et al., 2008). In adolescence significant changes to social relationships will happen. In particular, the transition to the university needs more and more independence from the family and the development of a new social network. Data showed that there was a positive relationship between the utilization of social support and self-esteem of university students (Peng et al., 2003; Ma, 2005). Moreover, it is suggested by some authors that during early adolescence the peer relationships take on increased importance (Furman and Buhrmester, 1992). The results of previous studies (Fan and Fang, 2004; Lian et al., 2016) showed that support from the peer group significantly influences the self-esteem of adolescents, especially the emotional support positively predicted the level of self-esteem.

Further data demonstrated that adolescents’ social relations outside the family might be a moderator for the connection of relationships with parents and adolescent adjustment. For example, Beyers and Seiffge-Krenke (2007) longitudinal study found that adolescents’ relationships with parents influenced later problems of behavior when interacting with friends and romantic partners. To our knowledge, no studies exist which examine the moderator effect of social support on the relationship between family function and self-esteem. Therefore, in the current study the relationship between the three factors was explored. The aim of this study is to examine the difference in family functioning and self-esteem between university students with and without grandparenting experience and to investigate the moderating effect of social support in the relationship between family functioning and self-esteem based on retrospective data from a group of university students.

### Hypothesis

First, we assumed that grandparenting experience would have influence on family functioning and level of self-esteem. Second, we assumed that social support would have moderating effect in the relationship between family functioning and self-esteem in the students with grandparenting experience; Third, from a perspective of moderation model, we hypothesized that in the relationship between family function and self-esteem it would show a significant difference between persons with high social support and low social support.

## Materials and Methods

### Participants

A sample of 2530 freshmen and sophomores (1372 were males, 1158 were females) was recruited at a university in Shanghai, a large city in the eastern part of China. The students’ ages ranged from 16 to 20 years, with an average of 18.79 years (*SD* = 0.02). Written informed consent was obtained from all the participants prior to the study. The written informed consent from the caretakers on behalf of the participants under 18 years was also obtained.

### Measures

#### Family Assessment Device (FAD)

The Family Assessment Device (FAD) is a well established measure to assess family functioning based on the theoretical concept of the McMaster Model of Family Functioning (MMFF). The term “family functioning” refers to the ability of the family to work together as a unit to satisfy the basic needs of its members (Ryan and Keitner, 2009). The MMFF emphasizes the interrelatedness of the family members and the family system, and represents six dimensions of family functioning that are considered of high relevance in clinical practice. It is made up of seven scales that assess six dimensions of the MMFF as well as the General Family Functioning. Among the six dimensions there are Problem Solving (PS), Communication (CM), Roles (RL), Affective Involvement (AI), Affective Responsiveness (AR), and Behavioral Control (BC) (Shek, 2001): (1) problem solving (the capability of the family to cope with problems in order to keep effective family functioning); (2) CM (the way of exchanging information between family members); (3) RL (whether the family assign certain tasks to guarantee implementation of family functions); (4) AR (to which extent the family members emotionally react to stimulation); (5) AI (to which extent the family members show concern to each other); and (6) behavior control (the behavioral models that the family establishes to cope with stressful situations). The instrument consists of 60 statements about a family; the requirement is to rate to which extent the description of each statement accords with their own family. All items are rated on a 4-point Likert scale, with answer choices from “1 = strongly agree” to “4 = strongly disagree.” Sample items for example include, “You can’t tell how a person is feeling from what they are saying,” “If someone is in trouble, the others become too involved,” “Making decisions is a problem in our family,” and, “We confront problems involving feelings.” The higher scores the respondents rate, the worse levels of family functioning indicate. The validity and reliability of the Chinese FAD has been demonstrated by Shek (2001, 2002). The test-retest reliability is 0.53–0.81, and coefficient alpha ranges from 0.53 to 0.94.

#### Rosenberg Self-esteem Scale

The Rosenberg Self-esteem Scale (SES; Rosenberg, 1965) was applied to assess self-esteem. This instrument consists of 10 items. It is a self-report measure of global self-esteem. All items are rated on a 4-point Likert scale with answer choices from “1 = strongly disagree” to “4 = strongly agree.” Sample items for example include “I am able to do things as well as most other people.” and “I take a positive attitude toward myself.” The total scores range from 10 (low level of self-esteem) to 40 (high level of self-esteem). Good levels of reliability and validity of the Chinese RSES have been demonstrated in the previous studies (Zhao et al., 2012; Kong and You, 2013). In this study, the Cronbach alpha coefficient for the RSES was 0.84.

#### Perceived Social Support Scale

The Perceived Social Support Scale (PSSS) was developed by Zimet et al. (1988). The scale consists of 12 items designed to assess perceived social support from three sources: Family, Friends, and Significant Others. Items are scored on a 7-point rating scale ranging from 1 (very strongly disagree) to 7 (very strongly agree) with possible scores ranging from 12 to 84. The current total perceived social support status can be measured according to the total score for the 12 items. The Chinese version of this scale has been widely used and exhibited good level of validity and reliability (Wang et al., 1999; Kong and You, 2013). In this study, the Cronbach’s a coefficient for the PSSS was 0.72.

#### Grandparenting Experience

The definition of grandparenting experience in this study refers to that individual during their childhood left their parents and was raised by grandparents for at least 1 year. The grandparenting experience was evaluated using a questionnaire designed by the authors that included sex, age, whether left parents and was raised by others in the childhood, the duration and caregiver. The students who reported that they left their parents and were raised by grandparents for at least 1 year during their childhood were defined as grandparenting group.

### Procedure

All of the participants volunteered to participate at the end of the mental health education course. They signed an informed consent form at first. Participants were instructed to complete a questionnaire survey including measures of family functions, self-esteem, and perceived social support as well as grandparenting information in a quiet classroom environment after informing consent. It took approximately 20 min for the students to complete all the instruments.

### Data Analysis

All statistical procedures were conducted using the Statistical Package for the Social Sciences (SPSS 17.0). Independent sample *t*-test was used to compare the level of self-esteem, family function score and social support score between the two groups with and without grandparenting experience; Pearson correlation was calculated to explore how levels of self-esteem and family functions as well as perceived social support were related; Hierarchical regression analysis was applied to examine the moderating effect of social support on the relationship between family function and self-esteem.

## Results

### Demographic Background of the Participants

Six hundred and forty-five (25.69%) students reported that they left their parents and were raised by grandparents for at least 1 year during their childhood; 1764 (70.25%) students were brought up only by their parents; and 121 (4.06%) students reported that they had experience of being raised by other persons, who were not their own parents or grandparents. Among the students who were only raised by their parents the percentage of only children was higher compared to the grandparenting group (80% vs. 74.1%; *X*^2^ = 9.648, *P* = 0.002); there was no statistic difference in proportion of gender between two groups (Parents-raising: male 53.5% vs. Grandparenting: male 57.1%; *X*^2^ = 2.463, *P* = 0.117).

### Comparison of the Level of Self-esteem, Family Function Scores and Social Support Scores between the Two Groups with and without Grandparenting Experience

The students with grandparenting experience scored lower on self-esteem and total score of social support as well as support from family compared to the students raised only by their parents, while on the sub-scales of family function such as CM, RL, AR, AI, and GF the grandparenting group scored higher than their counterpart group (see **Table 1**).

**Table 1:** Comparison of the level of self-esteem, social support and family function scores between two groups $\left(\overset{¯}{x}\pm s\right)$.

Variables	Grandparenting group (*n* = 645)	Parents-raising group (*n* = 1764)	*t*-value	*P*-value
**Self-esteem**	29.90 ± 4.256	30.28 ± 4.132	2.000	0.046
**Social support**				
Total	66.75 ± 10.523	67.90 ± 10.319	2.399	0.017
Family	22.41 ± 4.342	23.05 ± 4.127	3.3.15	0.001
Friends	22.39 ± 4.143	22.61 ± 3.979	1.190	0.234
Others	21.94 ± 3.948	22.21 ± 3.805	1.544	0.123
**Family function**				
PS	12.56 ± 2.091	12.38 ± 2.260	-1.821	0.069
CM	19.04 ± 3.847	18.59 ± 3.921	-2.500	0.012
RL	23.22 ± 3.711	22.77 ± 3.598	-2.681	0.007
AR	13.10 ± 2.923	12.82 ± 3.032	-2.010	0.045
AI	14.30 ± 2.798	13.89 ± 2.785	-3.229	0.001
BC	20.24 ± 2.755	20.14 ± 2.715	-0.780	0.436
GF	22.29 ± 4.942	21.61 ± 4.877	-2.996	0.003


*PS, problem solving; CM, communication; RL, role; AR, affective responsiveness; AI, affective involvement; BC, behavioral control; GF, general functioning.*

### Correlation between Family Function, Social Support and Self-Esteem

In both of the two groups there are obviously significant positive correlations between self-esteem and social support and obviously significant negative correlations between self-esteem and all the sub-scales of family function. The sub-scales of family function are positively inter-correlated (see **Table 2**).

**Table 2:** Correlation between family function, social support and self-esteem in two groups (r).

Variable	SS		PS		CM		RL		AR		AI		BC		GF	
								
	G	P	G	P	G	P	G	P	G	P	G	P	G	P	G	P
PS	-0.355^∗∗^	-0.394^∗∗^														
CM	-0.416^∗∗^	-0.461^∗∗^	0.510^∗∗^	0.581^∗∗^												
RL	-0.338^∗∗^	-0.425^∗∗^	0.410^∗∗^	0.439^∗∗^	0.579^∗∗^	0.604^∗∗^										
AR	-0.405^∗∗^	-0.442^∗∗^	0.377^∗∗^	0.449^∗∗^	0.669^∗∗^	0.651^∗∗^	0.535^∗∗^	0.543^∗∗^								
AI	-0.320^∗∗^	-0.401^∗∗^	0.339^∗∗^	0.344^∗∗^	0.603^∗∗^	0.576^∗∗^	0.627^∗∗^	0.636^∗∗^	0.488^∗∗^	0.541^∗∗^						
BC	-0.237^∗∗^	-0.263^∗∗^	0.290^∗∗^	0.323^∗∗^	0.269^∗∗^	0.325^∗∗^	0.434^∗∗^	0.451^∗∗^	0.341^∗∗^	0.345^∗∗^	0.393^∗∗^	0.406^∗∗^				
GF	-0.491^∗∗^	-0.524^∗∗^	0.613^∗∗^	0.630^∗∗^	0.753^∗∗^	0.765^∗∗^	0.668^∗∗^	0.662^∗∗^	0.643^∗∗^	0.673^∗∗^	0.688^∗∗^	0.675^∗∗^	0.415^∗∗^	0.395^∗∗^		
SE	0.297^∗∗^	0.384^∗∗^	-0.263^∗∗^	-0.310^∗∗^	-0.334^∗∗^	-0.382^∗∗^	-0.361^∗∗^	-0.380^∗∗^	-0.318^∗∗^	-0.314^∗∗^	-0.241^∗∗^	-0.354^∗∗^	-0.247^∗∗^	-0.264^∗∗^	-0.327^∗∗^	-0.399^∗∗^


*G, grandparenting group; P, parent-raising group; SS, social support; SE, self-esteem.*

*^∗^*P* < 0.05, ^∗∗^*P* < 0.01.*

### Hierarchical Regression Analysis on the Moderating Effect of Social Support on the Relationship between Family Function and Self-esteem

In order to test the moderating effects of social support on the relationships between family function and self-esteem, hierarchical regression procedures were performed as recommended by Baron and Kenny (1986). Before testing the moderating effects, the two predictor variables (social support and family function) were standardized to reduce problems associated with multicollinearity between the interaction term and the main effects (Frazier et al., 2004). Thus, *z*-scores were calculated for social support and family function.

The hierarchical multiple regression analyses were conducted in each group. In the hierarchical regression model, the order of entry was as follows. At step 1, the control variables (age, gender, and “only child status”) were entered into the regression equation. At step 2, the predictor variable (all sub-scales of family function) was entered with the method of stepwise (α = 0.05) into the regression equation. At step 3, the moderator variable (social support) was entered into the regression equation. At step 4, the interaction of each dimension of family function × social support was added with the method of stepwise (α = 0.05). Significant change in *R*^2^ for the interaction term indicates a significant moderator effect.

The results exploring predictors of self-esteem showed that for the grandparenting group three variables – RL (β = -0.170, *P* < 0.01), social support (β = 0.243, *P* < 0.01), and BC (β = -0.078, *P* < 0.05) had a significant effect on the self-esteem. In this model, lower family function score and higher social support score were associated with higher self-esteem score. Most importantly, there was a significant interaction between general family functioning and social support (β = -0.123, *P* < 0.01). These results suggest that social support moderated the relationship between general family functioning and self-esteem. In the parent-raising group, six significant predictors of self-esteem were found, these were social support (β = 0.212, *P* < 0.01), RL (β = -0.115, *P* < 0.01), CM (β = -0.111, *P* < 0.01), AI (β = -0.080, *P* < 0.01), BC (β = -0.065, *P* < 0.01), and PS (β = -0.062, *P* < 0.05). However, there was no significant interaction between family function and social support. Results of these analyses are presented in **Table 3**.

**Table 3:** Hierarchical regression analysis predicting self-esteem from family function and social support.

Group	Variables	*R*^2^	Δ *R*^2^	*F*	β	*t*
**Grandparenting**	Gender	0.000	0.000		-0.114	-3.329^∗∗^
	Age	0.000	0.000		0.034	1.026
	Only child status	0.010	0.010	2.502	0.029	0.868
	RL	0.139	0.129	29.244^∗∗^	-0.170	-3.839^∗∗^
	AR	0.165	0.026	28.494^∗∗^	-0.076	-1.610
	BC	0.177	0.012	22.144^∗∗^	-0.078	-2.120^∗^
	CM	0.182	0.005	19.949^∗∗^	-0.080	-1.624
	SS	0.209	0.027	23.677^∗∗^	0.243	6.059^∗∗^
	GF × SS	0.222	0.013	22.773^∗∗^	-0.123	-3.536^∗∗^
**Parent-raising**	Gender	0.000	0.000		-0.069	-3.182^∗∗^
	Age	0.000	0.000		-0.048	-2.195^∗^
	Only child status	0.017	0.017	9.870^∗∗^	0.013	0.588
	RL	0.186	0.169	96.862^∗∗^	-0.115	-3.678^∗∗^
	CM	0.195	0.009	82.203^∗∗^	-0.111	-3.500^∗∗^
	BC	0.201	0.006	71.005^∗∗^	-0.065	-2.648^∗∗^
	PS	0.203	0.002	61.758^∗∗^	-0.062	-2.296^∗^
	AI	0.206	0.003	55.153^∗∗^	-0.080	-2.664^∗∗^
	SS	0.237	0.031	66.069^∗∗^	0.212	8.386^∗∗^


*^∗^*P* < 0.05, ^∗∗^*P* < 0.01.*

*SS, social support; PS, problem solving; CM, communication; RL, role; AR, affective responsiveness; AI, affective involvement; BC, behavioral control; GF, general functioning.*

**FIGURE 1:**
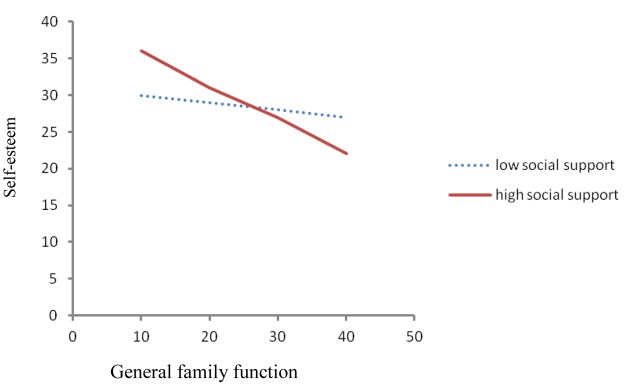
Effects of general family function and social support on self-esteem scores.

To illustrate the general family functioning × social support interaction for self-esteem in the grandparenting group, we plotted the regression of self-esteem on general family functioning at high and low levels of social support (see **Figure 1**). According to the procedures posed by Aiken and West (1991), we used the simple slope for the regression of self-esteem on general family function by using the high (one standard deviation above the mean) and low (one standard deviation below the mean) values for social support. There was a significant negative relation between general family functioning and self-esteem at high levels of social support (β = -0.452, *P* < 0.01). However, at low levels of social support, the relation between general family functioning and self-esteem was non-significant (β = -0.096, *P* > 0.05). Thus, among students with high social support, general family functioning was a significant determinant of self-esteem. In contrast, at low levels of social support, general family function did not influence self-esteem.

## Discussion

The current study was designed to examine the difference in family functioning and self-esteem between university students with and without grandparenting experience and the important role of social support on the relationship between family function and self-esteem in a sample of Chinese university students. We found that the students who have the experience of grandparenting showed a lower level of self-esteem than the students raised by their parents. Grandparents raising children is a common phenomenon in China, which is related to the country’s social and cultural background. The parents are both too busy to take care of the child or they are working in another city far away from their hometown, so the child is sent back to the grandparents to be raised until he or she goes to kindergarten or school (Wang, 2012). Chinese culture is collectivist and deeply influenced by Confucianism, while many western cultures are more individualistic. Collectivism emphasizes common interests, conformity, cooperation, and interdependence. These cultural traits strengthen extended families and collectives where each person takes responsibility for fellow members of their group (Hofestede, 2001). Therefore, in China many grandparents in retirement take over the responsibility of raising the grandchildren by their own choice. Song (2004) found that for late adolescents, parental attachment contributed significantly to both self-liking and self-competence. According to Bowlby (1982), the period from birth to 3 years old is crucial to establish attachment between the infant and main caregiver; individual’s fundamental self-perception and self-evaluation derive from the experience of interaction between the child and caregiver in the early childhood. The stable and health parent-child relationship makes the child feel that he or she is a favorite and capable person, and otherwise the unstable parent-child relationship makes the child feel unwelcome and worthless. The grandparenting experience could result in failure of establishment of secure attachment, and hence exert a negative influence on the self-esteem development. The children who left their parents and were raised by grandparents in infancy fail to establish an attachment relationship with their parents in their early childhood; instead they may become more attached to their grandparents. However, the lack of parental care and companionship means that when they came back to live with their parents, both the parents and the children would feel distant from each other, and the children could fail to gain strong sense of security and belonging, which might affect their self-awareness and self-evaluation (Song, 2004). Furthermore, the rearing style of grandparents is different from the parents; the grandparents may look after the grandchildren too carefully to give them enough exploration space; according to Erikson’s psychosocial stages, it would hinder their development of sense of autonomy and self-worth.

In addition, compared to their counterpart group the grandparenting group reported worse family function in terms of role, CM, AI as well as AR and general family function. In non-grandparenting families, the children and parents can develop a close parent-children relationship and the children can establish a good sense of trust and security in the core family, whereas in grandparenting families, the parents may be both too busy to take care of the child or they are working in another city far away from their hometown, they cannot fully take over the responsibilities of rearing the child. In that case the grandparents instead bear a certain responsibility of raising and educating the grandchildren, it is therefore possible that the children fail to develop a close attachment relationship to their parents. As a result, this could lead to less emotional exchange and low confidence between parents and children when the children came back to their core family. Li (2010) has investigated the parent-child relationship in the college students with grandparenting experience, it was found poor intimacy, lack of trust on each other and deficient initiative CM. Therefore, worse family function might be exhibited within the grandparenting family compared to the families where the children are raised by their parents.

The students with grandparenting experience also reported lower perceived social support than the students raised by their parents. Perceived social support can be understood as a personal subjective evaluation that he or her social network will provide effective help when needed (Lakey and Scoboria, 2005). It can be distinguished from the received support, which means that actual support is provided within a specific time frame (Uchino, 2009). Family support is an important part of social support. Compared to the students raised by their parents, the perceived family support of students with grandparenting experience could be generally lower due to their distant parent-child relationship.

As we expected, the results of correlative analysis showed that self-esteem had a significant positive relationship with social support and a negative relationship with family function. These results accord with previous studies on the relationships between self-esteem and social support (e.g., Goodwin et al., 2004; Birndorf et al., 2005; Zhang, 2007; Denissen et al., 2008; Lian et al., 2016; Chen et al., 2017) and family function (e.g., Heaven and Ciarrochi, 2008; An et al., 2010; Guo, 2012; Yen et al., 2013).

The most important finding of this study is that social support moderated the influence of general family function on self-esteem of the students with grandparenting experience. When these students reported a high level of social support, those with a low score of general family function reported a higher level in self-esteem. However, there were no differences in self-esteem between high and low general family function scores when they were confronted by a low level of social support. These results suggest that among the students who perceived great social support, more positive the family functioning were evaluated, higher level of self-esteem the students showed; whereas among the students who perceived poor social support, family functioning exert slight influence on the level of self-esteem. It seems that in the current study for the first time to report that social support influences on the association between general family function and self-esteem of students with grandparenting experience. We suggest that the results of our study can be ascribed to the self-development connected to the extent of early attachment. According to attachment theory, the children start to establish their basic self representation in reaction to the accessibility and sensitivity of parents and other caregivers in childhood, and these concepts are revised in the whole life (Bowlby, 1982; Bretherton, 1991). Therefore, if the caregiver is sensitive and accessible to the child, the child’s self model is valuable and worthy of being loved. On the contrary, if the parents are not available and sensitive to the child, the child’s self model is invaluable and is unworthy of being loved. Studies generally supports the idea that positive self-concept, including high levels of self-esteem and self-efficacy are associated with secure attachment to parents in infancy, childhood, and adolescence (Thompson, 1999; Arbona and Power, 2003). In Zhao’s (2011) study it was found that the proportion of secure attachment among the children with grandparenting experience was less than that of the children raised by their parents, instead the disorganized relationship was more. Those children who were often separated from their parents in early childhood to be raised by their grandparents, missed this crucial period during childhood to build a long-lasting attachment with their parents. Although the children may establish secure attachment with the grandparents, when they came back to their parents, they tended to feel a lack of care from their parents, they did not feel loved, and this kind of thought probably made the children feel unworthy (Li, 2010).

It is supposed that support from family, peer groups, and significant others, like teachers, or romantic partners for example is especially important for fostering identity and self-development of the college students who exhibit a lack of secure attachment to their parents (Allen and Land, 1999; Hazan and Zeifman, 1999; Fass and Tubman, 2002; Furman et al., 2002). The senses of self-importance, achievement and power gaining from the life experience are necessary for the construction of self-esteem (Yang and Zhang, 2003). If the college students are exposed to stressful situations, support from others might enable them to feel acceptance and strength, and thus enhance self-confidence and eventually lead to a higher self-esteem. Therefore, those students with a perceived lower social support as a result for being raised by their grandparents for at least 1 year, though reporting greater family function, will have difficulties in enhancing self-esteem.

Social support didn’t show moderating effect in the relationship between family function and self-esteem among the students without grandparenting experience, the possible reason could be that in the parents-raising family the influence of the parents may play more crucial role in the development of self-esteem of the children across the lifespan from infancy to adolescence than that of other family members as well as significant others outside of the family.

## Conclusion

In the current study some limitations should be mentioned. Firstly, in this study we used self-report measures to collect data, so it could have a threat to internal validity. We suggest using multiple methods for evaluation (e.g., peer reports) in the future study, which in contrast might reduce the effect of subjectivity. The second limitation lies in quantitative methodology of the study, in which it is difficult to make deep analysis of the impact of grandparenting experience on attachment and hence on self-esteem. In future research we suggest using experimental studies or qualitative studies to test the moderating model. The third limitation is that this sample came from a university population, so it limits the extent to which these findings could be generalized to other educational environments and geographic locations in China. Despite these limitations, there are important contributions of this study.

In conclusion, all the three hypotheses in this study are fully supported. The current study makes a significant contribution to the study of the family functioning and self-esteem in youth with grandparenting experience. It provides an empirical framework for researchers through testing the moderating effects of social support between family function and self-esteem in a sample of Chinese college students. The findings show that, social support is an important contributor in the relationship between family functioning and self-esteem in the students with grandparenting experience. Support from family members and significant others outside of the family are especially important for fostering identity and self-development of the college students who exhibit a lack of secure attachment to their parents. In future studies, first, more exploration may be needed of how the quality of early attachment and the time of the grandparenting (e.g., infancy, toddlerhood, early childhood, or middle childhood) influence the self-esteem of the students with grandparenting experience; second, further investigation may be need of how they develop friendships with peers and significant others that helps them improve self-esteem and efficacy. In addition, these findings may provide empirical reference to help to establish effective psychosocial interventions with the aim of improving social support and family function in university students with low self-esteem, and hence improving mental health status.

## Ethics Statement

This study was approved by the Institutional Review Board of Tongji University. All participants provided written informed consent prior to the study. For the participants under 18 years old, one parent provides written consent.

## Author Contributions

JS contributed to study design, recruitment of participants, data analysis and interpretation and writing of the manuscript. LW contributed to recruitment of participants and interpretation of results. YY and NS contributed to recruitment of participants. XZ contributed to study design and interpretation of results. CZ contributed to recruitment of participants. All authors have approved the final manuscript.

## Conflict of Interest Statement

The authors declare that the research was conducted in the absence of any commercial or financial relationships that could be construed as a potential conflict of interest.
